# Girls’ Stuff? Maternal Gender Stereotypes and Their Daughters’ Fear

**DOI:** 10.3389/fpsyg.2021.741348

**Published:** 2022-01-06

**Authors:** Antje B. M. Gerdes, Laura-Ashley Fraunfelter, Melissa Braband, Georg W. Alpers

**Affiliations:** Department of Psychology, School of Social Sciences, University of Mannheim, Mannheim, Germany

**Keywords:** gender differences, specific phobia, fear of snakes, anxiety in children, gender stereotypes, sex differences, social learning, gender roles

## Abstract

One of the most robust findings in psychopathology is the fact that specific phobias are more prevalent in women than in men. Although there are several theoretical accounts for biological and social contributions to this gender difference, empirical data are surprisingly limited. Interestingly, there is evidence that individuals with stereotypical feminine characteristics are more fearful than those with stereotypical masculine characteristics; this is beyond biological sex. Because gender role stereotypes are reinforced by parental behavior, we aimed to examine the relationship of maternal gender stereotypes and children’s fear. Dyads of 38 mothers and their daughters (between ages 6 and 10) were included. We assessed maternal implicit and explicit gender stereotypes as well as their daughters’ self-reported general fearfulness, specific fear of snakes, and approach behavior toward a living snake. First, mothers’ fear of snakes significantly correlated with their daughters’ fear of snakes. Second, mothers’ gender stereotypes significantly correlated with their daughters’ self-reported fear. Specifically, maternal implicit gender stereotypes were associated with daughters’ fear of snakes and fear ratings in response to the snake. Moreover, in children, self-reported fear correlated with avoidance of the fear-relevant animal. Together, these results provide first evidence for a potential role of parental gender stereotypes in the development and maintenance of fear in their offspring.

## Introduction

### Sex Differences in Specific Phobia

The most prominent and robust finding in anxiety disorders is that they are twice as common in women compared to men (30.5–33 vs. 19–22%, Kessler et al., 1994; McLean et al., 2011). This ratio was also replicated for specific phobias (26.5 vs. 12.9%, Frederikson et al., 1996), and differences in prevalence rates are especially pronounced for fear of animals, as approximately 12% of women but only 3% of men report clinically relevant animal phobia (Frederikson et al., 1996). Sex differences in the prevalence of anxiety disorders emerge already early in childhood (e.g., King et al., 2000; McLean and Anderson, 2009) and continue throughout young adulthood (Mackinaw-Koons and Vasey, 2000; Muris et al., 2000). Again, differences in prevalence rates between girls and boys are most pronounced for animal phobia, as girls are at a higher risk of acquiring animal fears than boys (odds ratio: 2.03, Meltzer et al., 2009).

Interestingly, especially animals such as spiders and snakes are feared more strongly by women than by men (Frederikson et al., 1996). Generally, fear of snakes belongs to the most frequent fears worldwide (Agras et al., 1969; Curtis et al., 1998; Depla et al., 2008). The percentage of people with ophidiophobia, meaning a clinically relevant fear of snakes, is estimated 2–3% in the population. Several studies found women to report fear of snakes twice as often compared to men and women consistently reach higher scores on questionnaires assessing symptoms of fear of snakes (Frederikson et al., 1996; Polak et al., 2016; Zsido, 2017).

### Theories Explaining Sex Differences in Fear and Anxiety: Biological Perspective

Previous research has focused mainly on biological explanations for sex differences in prevalence rates and demonstrates that genetic and evolutionary factors determine these differences to a certain extent (for an overview on different theories, see, e.g., Craske, 2003; McLean and Anderson, 2009). From the evolutionary perspective, error theory proposes that underestimating threat used to be more costly for women (and their offspring) compared to men (Haselton and Buss, 2000; Nesse, 2001, 2019). Thus, it has proven advantageous for the survival of women and their children to react fearful to potentially dangerous animals, such as snakes. However, fear of snakes and the sex-specific ratio still persist, even though snakes are actually harmless in some parts of the world, such as Western Europe (McLean and Anderson, 2009; Rakison, 2009). This persistence is likely driven by (phylo-) genetic factors (Möller et al., 2013). However, heritability accounts for up to one-third of the total variance of the development of specific phobias (for an overview of genetic influences, see Van Houtem et al., 2013), implying that also other factors play an important role.

### Theories Explaining Sex Differences in Fear and Anxiety: Socialization Perspective

Beyond biological explanations, it is discussed that social and cultural socialization factors contribute to sex differences in fear and anxiety disorders (McLean and Anderson, 2009; Murray et al., 2009; Debiec and Olsson, 2017), as well as specific phobias (Frederikson et al., 1996; Rakison, 2009). More specifically, gender role orientation and gender role socialization are thought to play a role for the development of anxiety and its sex differences (Yang et al., 1995). Gender role orientation describes the degree to which one identifies with traditional gender conceptions and the associated personal attitudes, self-concepts, social behaviors, and career choices. It is distinct from gender itself and is conceived as dynamic and multicausal (Livingston and Judge, 2008; Pérez-Quintana et al., 2017). According to social learning theories, children learn what is supposedly appropriate for their biological sex through a process of vicarious learning, thus developing gender stereotypes over time (Endendijk et al., 2013). Gender stereotypes are defined as beliefs about characteristics, behaviors, and roles typical for women and men (Endendijk et al., 2013).

### Development of Gender Role Orientation and Gender Stereotypes

According to theories on the development of gender roles (e.g., Bem, 1981), girls and boys were mainly socialized to develop gender-specific feminine and masculine behaviors and skills, respectively. Indeed, infants are already able to distinguish between male and female characteristics, such as voice or face, providing the basis for the formation of gender role stereotypes in 1-year-olds (Martin et al., 2002; Leaper and Friedman, 2007). Between 3 and 6, knowledge about one’s own gender and the gender of others consolidates and children start to form gender stereotypes (e.g., girls play with dolls, Maccoby, 2000; Leaper and Friedman, 2007). By elementary school, they have extensive gender knowledge, and rigid ideas about what males and females should be like and what does and does not fit the two sexes. This rigidity peaks between the ages of 5 and 7, and children’s gender stereotypes only slowly begin to become more flexible thereafter (Trautner et al., 2005).

Gender role concepts are shaped by family, school, peers, and media, but especially by parents, which typically have the first significant influence on their children’s behavior and attitudes, and thus on the gender socialization of their offspring (Leaper and Friedman, 2007). Parents tend to reinforce playing with gender-typical toys and encourage gender-typical activities, such as household tasks and hobbies (Antill et al., 1996). When children behave contrary to traditional gender roles, their activities often receive little support from parents (Kane, 2006; Kollmayer et al., 2018).

In addition to parental behavior and other environmental influences, there is evidence that genetic aspects explain the expression of gender role conformity or gender (a)typical behavior in children to a certain extent, as shown by family and twin studies (e.g., Iervolino et al., 2005; Alanko et al., 2010; Polderman et al., 2018).

### Gender Role Orientation and Fear in Adults

Based on the findings that were mentioned above, traditional or stereotypical gender role expectations are thought to be an influential factor in the development of anxiety disorders, as anxiety and fear correspond more to the stereotypical role of females and not to the role of males. Thus, cautious and fearful behavior is tolerated or encouraged more in girls, whereas courageous and fearless behavior is expected and encouraged more in boys (McLean and Anderson, 2009). Moreover, this differential parental response to child behavior is probably more pronounced in parents who have more traditional gender stereotypes (Doey et al., 2014).

Evidence for a (direct) relationship between gender roles and anxiety comes from several studies with adults. For example, males and females with high femininity scores indicated higher (general) fear and anxiety levels compared to individuals rated as more masculine (Dillon et al., 1985). In a student sample, higher masculinity and lower femininity were associated with lower depression and anxiety symptoms in both, male and female students (Arcand et al., 2020).

For specific fear, males and females who rated themselves as more feminine were more fearful of all animals (Tucker and Bond, 1997), and this fear of animals was negatively associated with masculinity independent of the biological sex (Arrindell, 2000). In addition to self-report data, a few studies investigated the relationship between femininity/masculinity and behavioral markers of fear (McLean and Hope, 2010; Stoyanova and Hope, 2012). Results revealed that lower masculinity scores were associated with greater avoidance of a spider during the behavioral approach test, regardless of a biological sex (McLean and Hope, 2010). Similarly, a negative correlation between masculinity and anticipatory anxiety during approach was found in women, but not in men (Stoyanova and Hope, 2012).

### Gender Orientation and Fear in Children

Gender roles were also found to impact children’s anxiety. In 120 healthy male and female children between 6 and 12 years, gender role identity and attitudes, as well as the intensity of feelings toward peers as indexed by an Emotional Story Task, were assessed. Interestingly, girls reported higher levels of fear than boys and gender role identity accounted for more of the variance than the child’s biological sex. Thus, both sexes with higher scores on feminine gender role report higher levels of fears (Brody et al., 1990).

Furthermore, a non-clinical sample of 209 children between 10 and 13 years and their parents completed several questionnaires to assess gender role orientation, playing preferences, as well as fear and anxiety. An association between femininity, a preference for female activities and self-reported fear revealed that gender role orientation accounted for more of the variance in fear scores than the child’s sex (Muris et al., 2005). For healthy adolescents between 14 and 19 years, it was also shown that masculinity was negatively associated and femininity was positively associated with anxiety symptoms (Palapattu et al., 2006). In addition, in a sample of children between 9 and 13 years, gender role orientation mediated the relation between biological sex and anxiety sensitivity, supporting gender role orientation as an explanation for observed gender differences in anxiety (Stassart et al., 2014). Similarly, in a clinical sample of children with anxiety disorders, higher levels of masculinity were negatively associated with levels of fearfulness and specific fears independent of the biological sex (Ginsburg and Silverman, 2000).

### Current Research Question

In sum, there is strong evidence that gender role orientation and fear (behavior) are related within samples of adults, as well as within samples of children. Furthermore, it is assumed that parenting behavior is influenced, among others, by parental fears but also by gender role expectations and stereotypes (Doey et al., 2014).

However, research on the impact of parental gender stereotypes on children‘s fear is missing so far. Therefore, the present study aims to investigate this association with a special focus on mother–daughter dyads. To measure fear at different levels (Ollendick et al., 2011), we applied a behavioral approach test with a living snake to directly assess children‘s fear level and avoidance behavior in addition to different fear questionnaires from mothers and their daughters. For measuring gender role orientation and stereotypes, we administered questionnaires and a computer task with the mothers and children to include explicit and implicit measures of gender stereotypes.

We expect that daughters of mothers with gender role conforming attitudes show more fear than of mothers with less gender role conforming attitudes. This should be apparent in fear questionnaires, but also especially during the approach test.

## Materials and Methods

### Participants

The sample consists of *N* = 38 healthy girls at the age of 6–10 and their mothers. The mean age of the daughters was 7.66 years (*SD* = 1.28). The age of the mothers ranged from 27 to 52 years (*M* = 39.63, *SD* = 1.28). The sample comprises mothers with diverse educational backgrounds and professions. In large part, however, the mothers had university degrees (63.2%) and had an average of almost two children (*M* = 1.92, *SD* = 0.73). The majority of mothers reported that they spend most of the time with their children (84.2%), whereas 10.5% of the mothers reported that father and mother spend equal time with the children; 5.3% indicated that others, such as grandparents, spend the most time with the children. On average, the mothers reported to spend about 7 h per day with their daughters (*M* = 6.9, *SD* = 3.5).

### Measures

#### Daughters

##### Questionnaires

The daughters’ level of anxiety was measured with the phobia questionnaire for children [Phobiefragebogen für Kinder und Jugendliche (PHOKI), Döpfner et al., 2006], which is a German adaptation of the *Fear Survey Schedule for Children* (Ollendick, 1983). It consists of 96 items that measure fear of various objects and situations on a three-point response scale (0 = never, 1 = sometimes, and 2 = often). The sum score can range between 0 and 192, whereas a high sum score reflects a high level of anxiety. The children’s fear of snakes was measured following a multimodal approach. The *Snake Anxiety Questionnaire* (SNAQ, Klorman et al., 1974) was used as a measure of self-reported fear of snakes. It consists of 30 statements that can be answered with yes or no. The sum score can range between 0 and 30, whereby a higher sum score stands for stronger fear of snakes.

To assess daughters’ identification with gender roles, a short form of the *Children’s Sex Role Inventory* (CSRI, Boldizar, 1991) was used, which consists of 10 masculine, feminine, and neutral items each. The questionnaire measures masculinity and femininity. Responses were recorded on a four-point scale ranging from 1 = “does not apply to me at all” to 4 = “applies to me very much.” The CSRI is equivalent to the *Bem Sex Role Inventory* (Bem, 1974) for adults. The explicit gender stereotypes were measured with the *Gender-Stereotyped Attitudes Scale for Children* (GASC, Signorella and Liben, 1985). It consists of 32 questions with gender-stereotypical content (e.g., Who can fix a car?) that can be answered with “man,” “woman,” or “both.” The sum score is calculated from the number of items for which a child answers “both.” This sum score can range between 0 and 32, whereby higher scores reflect less gender-stereotypical thinking.

##### Behavioral Approach Test

To measure avoidance behavior, the daughters completed a *Behavioral Approach Test (BAT)*, in which they were instructed to approach a snake in a transparent box. The girls were asked to stand on a marked position around 2.5 m away from the box. The instructed task was to approach the box with the snake stepwise. The BAT consisted of the following five steps:

1. *Take one step toward the snake*.
2. *Take another step toward the snake*.
3. *Stand directly in front of the box with the snake inside*.
4. *Hold your hands above the box for 3 s*.
5. *Put your hands on the locked box*.

Completed steps were coded with one; uncompleted steps were coded with zero, which makes a maximum sum score of five for the approach behavior. With every step, the girls rated their current fear level on a scale ranging from 0 to 10 (0 = no fear, 10 = maximum fear). In general, the BAT is primarily used as a behavioral measure of fear in children. In a study with children at the age of 7–13, the retest reliability for the completed steps after an hour was *r* = 0.92 (Ollendick et al., 2011).

##### Implicit Measure

To assess daughters’ implicit gender stereotypes, the *Action Inference Paradigm* (AIP) were applied (Banse et al., 2010). In this paradigm, the participating child is instructed to help Santa Clause distribute gifts (by pressing the appropriate button) to a girl and a boy. The task starts with 20 practice trials with red and blue presents. These are followed by 32 congruent trials in which stereotypically female toys should be distributed to a girl and stereotypically male toys to a boy. In the subsequent incongruent trials, stereotypically female toys should be distributed to a boy and stereotypically male toys to a girl. During the task, reaction times are measured to determine the discrepancy between congruent and incongruent trials. Thus, the AIP reflects a child-adequate version of the Implicit Associations Test (IAT). The AIP-task was programmed and presented using *Presentation* (Neurobs, Inc., Albany, California, United States; www.neurobs.com).

#### Mothers

##### Questionnaires

The mothers’ level of anxiety was measured with 55 items of the *Fear Survey Schedule* (FSS, Hallam and Hafner, 1978). It measures fear of different objects and situations on a four-point response scale from 0 (“no fear”) to 3 (“extreme fear”). The sum score can range from 0 to 165. Higher sum scores indicate higher levels of fear.

Trait and state anxiety were assessed using the *State-Trait Anxiety Inventory* (STAI, Laux et al., 1981). Similarly to the daughters, the SNAQ was used to measure fear of snakes (Klorman et al., 1974).

In addition, the German version of the *Bem Sex Role Inventory* (BSRI, Bem, 1974; Schneider-Düker and Kohler, 1988), a questionnaire with 40 items to survey the gender-related self-concept, was answered by the mothers. Individuals can describe themselves regarding gender-typical characteristics on a seven-point scale from 1 (“the characteristic never applies”) to 7 (“the characteristic always applies”). The BSRI provides a femininity and masculinity scale.

In terms of the mothers’ gender stereotypes, the *Child-Rearing Sex-Role Attitude Scale* (CRSRAS, Burge, 1981) was used to assess explicit child-rearing sex-role attitudes. It consists of 28 items on a five-point response scale (from 0 = do not agree at all to 4 = fully agree), with a sum score between 0 and 112, whereby a higher sum score indicates low manifestation of explicit gender stereotypes.

##### Implicit Measure

To measure implicit gender stereotypes, a gender-career *Implicit Association Test* (IAT, Greenwald et al., 1998; Nosek et al., 2007) was applied. It assesses to what extent the participant associates female names with family-related words and male names with career-related words. To compute a participant’s score, practice trials were included, incorrect trials were excluded, and individual SDs were used (Greenwald et al., 2003). The IAT was also programmed and presented using *Presentation* (Neurobs, Inc., Albany, California, United States; www.neurobs.com).

### Procedure

The complete study protocol was approved by the Ethic Committee of the University of Mannheim, Germany (EK Mannheim 08/2018). The mother–daughter dyads were recruited *via* emails for primary schools and secondary schools in Mannheim and *via* press. After arriving at the laboratory, mothers and daughters were shown the experimental setups and were informed about the procedure. After that, informed consents were obtained from mothers and daughters. The study took part in three separate rooms. In one room, mothers first completed the IAT and then answered the questionnaires *via* SoSci Survey (Leiner, 2014). Meanwhile, the daughters answered the first two questionnaires (PHOKI, SNAQ) in a separate room with the help of a female experimenter. To hold their attention, the daughters completed the AIP at the computer before answering the remaining questionnaire (GASC, *CSRI*). To keep the variance due to differences in reading competency low, the experimenter read the questionnaires out to the girls. Finally, the experimenter and child entered a third room to perform the BAT. As a reward, the daughters received a certificate, sweets, and a toy. The mothers got a compensation for travel costs.

### Statistical Analysis

First, we conducted correlational analyses within and between mothers’ and daughters’ questionnaires, outcomes of implicit measures (AIP, IAT) and the BAT. Second, to predict daughters’ fear of snakes, as measured by fear ratings and number of steps during the behavioral approach test with the real snake (BAT), we entered all variables with a significant relationship to these independent variables into (multiple) linear regressions. For the correlations, we used Pearson’s correlations. Correlation coefficients between 0.1 and 0.3 can be interpreted as small or weak, coefficients between 0.3 and 0.5 as moderate, and coefficients above 0.5 can be interpreted as high effects (Cohen, 1988). All analyses were performed with SPSS-21 software, and hypotheses were tested with a two-sided significance level of 0.05. Due to the exploratory nature of this study, we refrained from correction for multiple testing (Streiner and Norman, 2011). Regarding the present sample size, *post hoc* power analyses were performed with G-Power (Faul et al., 2009) for significant correlations between maternal gender stereotypes and daughters’ fear indices, as well as for the linear regressions.

## Results

### Descriptive Data

#### Mothers

Mothers’ trait and state anxiety (trait anxiety: *M* = 37.03, *SD* = 9.87, state anxiety: *M* = 34.63, *SD* = 8.25), as well as their reported fear of snakes (*M* = 7.52, *SD* = 6.10) were in the normal range for women (Klorman et al., 1974; Laux et al., 1981). Similarly, the total sum score of the Fear Survey Schedule indicated a medium level of average fears (*M* = 25.24, *SD* = 10.84).

The mean score on the masculinity scale of the *Bem Sex Role Inventory* (BSRI) for the mothers was *M* = 4.60, *SD* = 0.69, and *M* = 4.77, *SD* = 0.52 on the femininity scale. The scores did not differ significantly, *t*(37) = 1.43, *p* = 0.16. Thus, on average, the level of femininity and masculinity was relatively balanced within our sample.

Similarly, the sum score of the CRSCR indicates a relatively low level of explicit sex-role attitudes with regard to their child-rearing (*M* = 102.97, *SD* = 7.81 – see Burge, 1981).

The implicit measure, reflected by the IAT-score, is on a medium level of implicit traditional gender stereotypes (*M* = 0.37, *SD* = 0.41, Nosek et al., 2007).

#### Daughters

Similar to their mothers, daughters’ fear of snakes (*M* = 7.89, *SD* = 6.54) and scores of the PHOKI (*M* = 47.66, *SD* = 20.84) were in the normal range (Ollendick, 1983). The mean score on the masculinity scale of the *CSRI* was *M* = 2.73 (*SD* = 0.48) and *M* = 3.19 (*SD* = 0.42) on the femininity scale. Comparing both scores revealed a slight predominance of femininity within the daughter sample, which is plausible for a female sample, *t*(37) = 6.79, *p* < 0.001.

Similarly, the explicit measure of children’s gender-stereotyped attitudes, assessed with the GASC, revealed comparatively low gender-stereotypical thinking in our sample (*M* = 17.71, *SD* = 6.47; see Signorella and Liben, 1985).

The mean score of the AIP^1^, reflecting implicit gender stereotypes, was *M* = 0.63 (*SD* = 0.32), showing that the reactions were significantly faster in stereotypical than in non-stereotypical trials, *t*(37) = 12.01, *p* < 0.001.

Regarding the BAT, the majority of the daughters (*n* = 32, 84.2%) completed all five steps of the test (mean number of steps *M* = 4.58, *SD* = 1.10). The overall mean fear rating during the BAT was relatively low (*M* = 2.51, *SD* = 3.36), whereas the fear rating sum during the BAT was in a medium range (*M* = 12.53, *SD* = 14.64 with a range from 0 to 50). However, there was an increase in fear ratings from step to step. For step 1, the mean fear rating was *M* = 1.53 (*SD* = 2.49) with one girl giving a fear rating of 10, while at step 5, fear ratings of *M* = 3.16 (*SD* = 3.94) were reported, with seven girls reporting a fear rating of 10.

### Correlational Analysis

#### Correlations Among Mothers’ Measures

Concerning the different explicit and implicit measures, we found significant correlations between mothers’ state and trait anxiety and their fear of snakes [STAI-state: *r*(38) = 0.387, *p* = 0.016; STAI-trait: *r*(38) = 0.453, *p* = 0.004]. Furthermore, trait anxiety and masculinity of the *BSRI* were moderately correlated, *r*(37) = −0.38, *p* = 0.018. Thus, trait and state anxiety were positively associated with specific fears, whereas higher levels of masculinity were associated with lower trait anxiety. Furthermore, there was a significant correlation of explicit child-rearing sex-role attitudes and fear of snakes, *r*(38) = −0.353, *p* = 0.030, indicating that more conservative sex-role attitudes in mothers are associated with higher fear of snakes. For all correlations, see Table 1.

**Table 1:** Correlations of measures among mothers.

	Fear measures				Stereotype measures		Sex role measures	
	STAI		FSS	SNAQ	CRSRAS	IAT	SRI	
	Trait	State					Femininity scale	Masculinity scale
**Fear measures**							
STAI							
Trait	-							
State	0.62^**^	-						
FSS	0.25	0.19	-					
SNAQ	0.45^**^	0.39^*^	0.31	-				
**Stereotype measures**							
CRSRAS	0.10	0.13	−0.26	−0.35^*^	-			
IAT	−0.04	0.16	0.29	0.23	−0.11	-		
**Sex role measures**							
BSRI							
Femininity scale	−0.23	−0.04	0.23	−0.27	−0.01	−0.15	-	
Masculinity scale	−0.38^*^	−0.24	−0.21	−0.31	0.14	0.09	0.25	-

** significant on the 0.01 level;

* significant on the 0.05 level.

SNAQ, Snake Anxiety Questionnaire; STAI, State-Trait Anxiety Inventory; FSS, Fear Survey Schedule; CRSRAS, Child Rearing Sex Role Attitude Scale; IAT, Implicit Association Test; and BSRI, Bem Sex Role Inventory. *N* = 38 (*n* = 37 for correlations with the AIP).

#### Correlations Among Daughters’ Measures

With regard to the daughters’ measures, there were significant correlations between general fearfulness (assessed by the PHOKI) and specific fear of snakes, *r*(38) = 0.483, *p* = 0.002. Most important, we found meaningful correlations between general fearfulness (PHOKI), fear of snakes (SNAQ), and daughters’ fear rating sum and avoidance behavior during the behavioral approach test [PHOKI and BAT fear rating: *r*(38) = 0.335, *p* = 0.040; SNAQ and BAT fear rating: *r*(38) = 0.626, *p* < 0.001; and BAT number of steps and SNAQ: *r*(38) = −0.496, *p* = 0.002]. This finding shows that self-reported fear of snakes reflects in higher fear ratings and avoidance behavior in the presence of a real snake. For all correlations, see Table 2.

**Table 2:** Correlations of measures among daughters.

	Fear measures				Stereotype measures		Sex role measures	
	PHOKI	SNAQ	BAT		GASC	AIP	CRSI	
			fear	steps			Femininity scale	Masculinity scale
**Fear measures**
PHOKI	-							
SNAQ	0.48^**^	-						
**BAT**								
Rating	0.34^*^	0.63^**^	-					
Steps	−0.12	−0.50^**^	−0.69^**^	-				
**Stereotype measures**								
GASC	0.06	0.03	−0.14	0.04	-			
AIP	0.00	0.07	−0.15	−0.07	0.25	-		
**Sex role measures**								
CRSI								
Femininity scale	−0.03	−0.16	−0.15	0.30	0.03	−0.26	-	
Masculinity scale	−0.12	0.08	0.09	0.05	0.03	−0.14	0.58^**^	-

** significant on the 0.01 level;

* significant on the 0.05 level.

PHOKI, Phobiefragebogen für Kinder und Jugendliche (German version of the Fear Schedule for Children); SNAQ, Snake Anxiety Questionnaire; BAT, Behavioral Approach Test; AIP, Action Inference Paradigm; GASC, Gender-Stereotyped Attitudes Scale for Children; and CSRI, Children’s Sex Role Inventory. *N* = 38 (*n* = 37 for correlations with the AIP).

#### Correlations Between Mothers’ and Daughters’ Measures

In the next step, we correlated the measures of daughters with measures of their mothers. As expected, there was a significant correlation between fear of snakes in mothers and their daughters, *r*(38) = 0.361, *p* = 0.026. The higher the reported fear of the mother, the higher the fear of the daughter.

Regarding the maternal implicit gender stereotypes, we found significant correlations between the IAT-derived implicit gender stereotypes and daughters’ fear of snakes, *r*(38) = 0.427, *p* = 0.009, as well as daughters’ fear rating sum during the behavioral approach test, *r*(38) = 0.344, *p* = 0.040. These correlations indicate that a greater extent of maternal implicit gender stereotypes is associated with higher fear levels of their daughter – for self-reported fear of snakes as well as for fear ratings during presence of a real snake. For all correlations, see Table 3.

**Table 3:** Correlations between measures of mothers and daughters.

	Daughters’ fear measures				Daughters’ stereotype measures		Daughters’ sex role measures	
	PHOKI	SNAQ	BAT		GASC	AIP	CRSI	
			fear	steps			Femininity scale	Masculinity scale
**Mothers’ fear measures**								
STAI								
Trait	−0.18	0.11	−0.23	0.09	0.03	−0.01	0.09	0.13
State	−0.11	0.17	−0.14	−0.00	0.13	−0.11	0.27	0.15
FSS	0.12	−0.00	−0.27	0.16	−0.04	0.20	−0.24	−0.04
SNAQ	0.11	0.36^*^	0.23	−0.31	−0.05	−0.11	−0.14	0.15
**Mothers’ stereotype**								
CRSRAS	−0.19	−0.16	−0.10	−0.02	0.24	−0.03	0.09	0.01
IAT	0.01	0.43^**^	0.34^*^	−0.32	−0.23	0.11	−0.42	0.04
**Mothers’ sex role**								
BSRI								
Femininity scale	0.06	−0.24	−0.20	0.11	−0.10	0.01	0.12	−0.07
Masculinity scale	−0.18	−0.21	0.05	−0.17	−0.29	−0.18	0.19	0.15

** significant on the 0.01 level;

* significant on the 0.05 level.

Children’s measures: PHOKI, Phobiefragebogen für Kinder und Jugendliche (German version of the Fear Schedule for Children); SNAQ, Snake Anxiety Questionnaire; BAT, Behavioral Approach Test; AIP, Action Inference Paradigm; GASC, Gender-Stereotyped Attitudes Scale for Children; and CSRI, Children’s Sex Role Inventory. Mothers’ measures: STAI, State-Trait Anxiety Inventory; FSS, Fear Survey Schedule; SNAQ, Snake Anxiety Questionnaire; CRSRAS, Child Rearing Sex Role Attitude Scale; IAT, Implicit Association Test; and BSRI, Bem Sex Role Inventory. *N* = 38 (*N* = 37 for correlations with the AIP).

*Post hoc* power analyses revealed the power to detect the given correlations between mothers’ gender stereotypes and the daughters’ fear of snakes before and during the BAT to be 0.81 and 0.6, respectively. To reach a satisfactory power of 0.8 for the correlation with fear ratings during the BAT, the sample size would have to increase to at least 61 dyads of mothers and daughters.

### Regression Analysis

According to the above-reported significant correlations, we conducted two linear regressions using daughters’ fear questionnaire scores (PHOKI, SNAQ) as predictors for their fear rating during the BAT and using the snake fear questionnaire (SNAQ) to predict their number of steps during the BAT. In a second step, we conducted two linear regressions to predict daughters’ fear of snakes (SNAQ) and fear ratings during the BAT with maternal measures. Here, we used maternal fear of snakes (SNAQ) and explicit stereotypes (IAT) as predictors for daughters’ fear of snakes and explicit stereotypes of the mothers (IAT) as predictor for daughters’ fear ratings during the BAT.

Fear ratings during the BAT were significantly predicted by the daughters’ SNAQ-score, *β* = 0.605, *t*(35) = 4.03, *p* < 0.001. The overall model explained a significant proportion of variance, corrected *R^2^* = 0.359, *F*(2, 36) = 11.35, *p* < 0.001.

The number of steps during the BAT was significantly predicted by the daughters’ SNAQ-score, *β* = −0.496, *t*(35) = 3.42, *p* = 0.002, also explaining a significant proportion of variance, corrected *R^2^* = 0.225, *F*(1, 36) = 11.72, *p* = 0.002.

**Figure 1:**
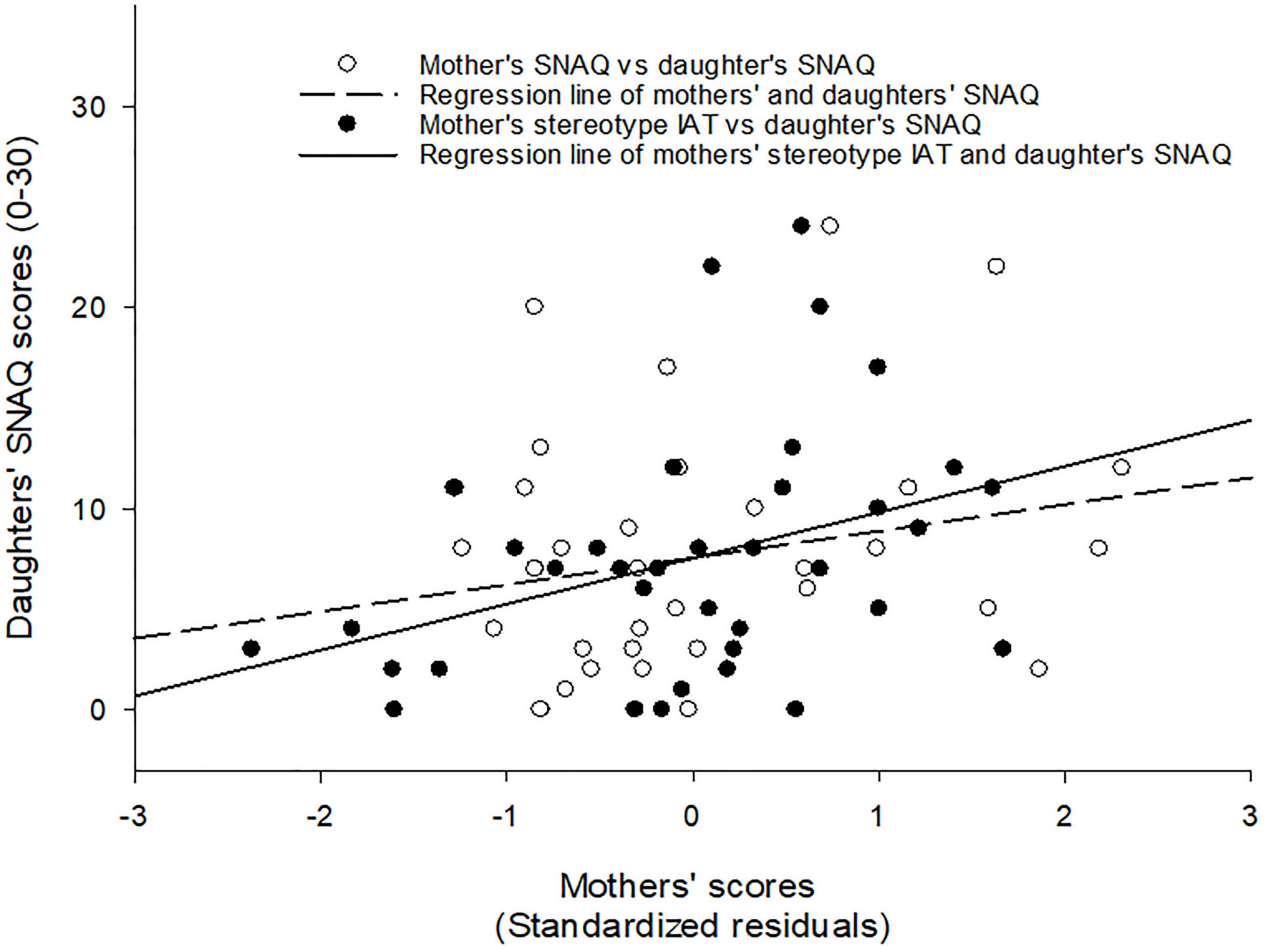
Scatter plot with fitted regression lines showing the association between mothers’ implicit gender stereotypes (IAT scores), mothers’ fear of snakes (SNAQ scores), and daughters’ fear of snakes (SNAQ scores). IAT, Implicit Association Task; SNAQ, Snake Questionnaire.

When daughters’ fear of snakes was predicted by maternal measures, it was found that maternal implicit stereotypes measured by the IAT were a significant predictor, *β* = 0.378, *t*(35) = 2.41, *p* = 0.022, whereas maternal snake fear was not significant, *β* = 0.220, *t*(35) = 1.40, *p* = 0.170. The complete model explained a significant proportion of variance, corrected *R^2^* = 0.182, *F*(2, 35) = 4.89, *p* = 0.014 – see Figure 1. Given this effect size, *post hoc* power analyses revealed a chance to detect this effect of 0.37. To reach a power of 0.8, a sample size of at least 99 dyads of mothers and daughters would be necessary.

**Figure 2:**
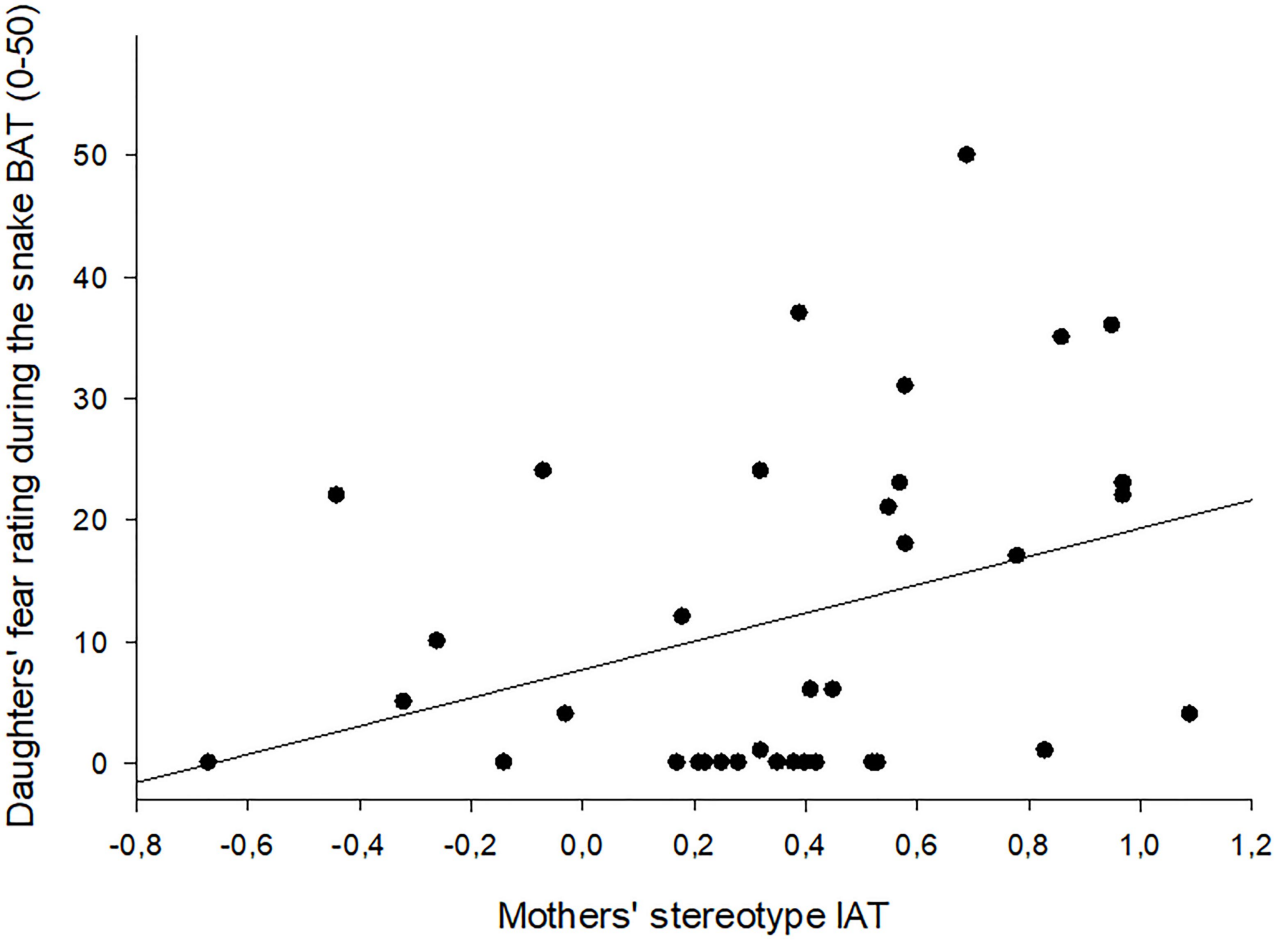
Scatter plot with fitted regression line showing the association between mothers’ implicit gender stereotypes (IAT scores) and daughters’ fear ratings during the snake BAT. IAT, Implicit Association Task; BAT, Behavioral Approach Test.

In addition, daughters’ fear rating during the BAT was also significantly predicted by maternal implicit stereotypes measured by the IAT, *β* = 0.344, *t*(35) = 2.14, *p* = 0.040. This model explained a significant proportion of variance, corrected *R^2^* = 0.092, *F*(1, 35) = 4.57, *p* = 0.040 – see Figure 2. The chance to detect this effect was found to be nearly satisfactory, given a power of 0.7. To ensure sufficient statistical power for this effect, a sample size of 47 dyads would be needed.

## Discussion

In clinical practice and across many studies, the prevalence of specific fears and phobias is much higher in girls, and their fear persists into adulthood (Frederikson et al., 1996; Polak et al., 2016; Zsido, 2017). Gender role orientation and gender stereotypes have been found to be important determinants of anxiety (Dillon et al., 1985; Arcand et al., 2020), as well as of sex differences in specific fears (Tucker and Bond, 1997; Arrindell, 2000; McLean and Hope, 2010; Stoyanova and Hope, 2012). However, very few studies have focused on the association between parent’s gender role stereotypes and children’s fear. Thus, the present study investigates whether maternal fears, gender role orientation, and specifically gender stereotypes are related to daughters’ level of self-reported general fearfulness, specific fear of snakes, as well as their behavior toward a living snake during a behavioral approach test.

Our results show that daughters’ general fear of snakes correlates with self-reported fear ratings and less approach behavior toward the fear-relevant animal during a behavioral approach test. Furthermore, mothers’ fear of snakes is significantly associated with their daughters’ fear of snakes. For the mothers, we found a negative association between masculinity and trait anxiety. Most important for the present research aim, maternal gender stereotypes were significantly associated with daughters’ self-reported fear. More specifically, maternal implicit gender stereotypes assessed with the IAT predicted daughters’ fear of snakes and fear ratings while approaching a living snake.

Therefore, our study shows first evidence that traditional gender role stereotypes in mothers are significantly associated with higher fear levels in their daughters. However, as this is one of the first studies with correlational evidence for an influence of maternal stereotypes on children’s fear, the exact underlying processes should be further investigated in future studies.

### Conformity With Previous Studies on Gender Roles

The main results are well in line with previous evidence showing that stereotypical gender roles can be significantly related to fear. For example, it has been reported for children and adults that higher fear levels are associated with higher levels of femininity and lower levels of masculinity independent of the biological sex (Ginsburg and Silverman, 2000; Chaplin et al., 2005; Muris et al., 2005). As an underlying process, we assume that parents, which tend to think in a stereotypical manner, tolerate and reinforce anxiety-related behavior in their daughters more often and encourage daughters less to face anxiety-provoking situations (Chaplin et al., 2005). This distinctive parenting behavior could increase and maintain anxiety in daughters *via* verbal information or modeling, thus increasing differences in prevalence rates of (specific) anxiety between males and females (Muris and Field, 2010; Remmerswaal et al., 2013). Already in very young children, parents talk more with their daughters about emotional states with a focus on negative emotions compared to sons (Fivush et al., 2000). Similarly, there is evidence from research on gender differences in math anxiety, revealing that mothers specifically communicate (math) gender stereotypes to their daughters, which is further associated with enhanced math anxiety and affects academic preferences of the daughters (Batchelor et al., 2017). Interestingly, the influence of maternal stereotypes on children’s fear in our study could be shown only for the implicit measure of stereotypes. This finding seems plausible considering that implicit measures are assumed to reduce self-presentational biases compared to explicit measures – especially in assessing (gender) stereotypes (White and White, 2006). This assumption possibly also applies to our sample, as the stereotypes assessed by explicit measures are relatively low and do not correlate with the implicit measure.

Although we observed associations between mothers’ stereotypes and daughters’ fears, and it is likely that mothers’ stereotypes will have developed earlier, this is not proof of causality. Also, we cannot rule out possible genetic influences, such that gender orientation might be inherited to a certain extent from parents, which in turn might mediate the relationship between parental gender stereotypes and children’s fear. However, besides evidence that genetic influences on gender role orientation become apparent mainly at a later age (see Polderman et al., 2018), our findings show a direct association of maternal gender roles and fear in children and no significant association between child and maternal gender roles. Thus, the association with fear does not appear to be mainly mediated by child gender roles, at least in our study.

Plausible mechanisms for other mediating processes are modeling (Bandura et al., 1967) or instruction (Bublatzky et al., 2014). For the latter, we documented experimentally that threat instructions do not need to be elaborate to result in surprisingly stable specific fear responses. Interestingly, parents may or may not be aware of these influences. Future research will have to identify the targets of this intergenerational learning: Whether parents convey enhanced risk estimations (Hengen and Alpers, 2019) or avoidant behavioral tendencies (Pittig et al., 2014) is to be explored.

Importantly, our results may have relevant implications for fear prevention and treatment (Bekker and van Mens-Verhulst, 2007; Hallers-Haalboom et al., 2020), especially in girls, by considering (parental) gender role expectations and dispelling gender stereotypes. Interestingly, there is first evidence that courage can have anxiety-reducing effects and may counteract the development of pathological fears (Muris and Field, 2010). For example, dispositional courage is positively associated with enhanced approach behavior toward a living spider in spider fearful women (Cougle and Hawkins, 2013), and courage was able to predict approach behavior even after controlling for spider fear (Norton and Weiss, 2009). Similarly, higher levels of self-reported courage in schoolchildren were also related to lower anxiety levels (Muris and Field, 2010).

Thus, becoming aware of one’s own stereotypes and encouraging children – especially girls – to face challenging or unfamiliar situations could be one promising approach to prevent anxiety among girls and women. Moreover, these insights may be also relevant for the gold standard treatment for phobias, i.e., exposure therapy, by informing cognitive preparation of psychoeducation that typically precedes exposure to feared animals (Alpers, 2010).

## Conformity with Other Theories on Sex Differences in Anxiety

Several theories aim at explaining sex differences in prevalence rates of anxiety and phobias, specifically. However, comprehensive approaches integrating evolutionary, genetic, physiological, and social influences are scarce (e.g., Craske, 2003). In the following section, we check for the compatibility of our results with prominent theories on the acquisition of (sex differences) in fear of snakes.

Preparedness theory states that it has proven advantageous for the survival of humankind to react fearful to potential threat, such as snakes (Seligman, 1971). More than that, it implies that one single confrontation is sufficient to produce avoidance behavior (“Ease of Acquisition”) and proposes a higher resistance to extinction for such stimuli. Furthermore, the error detection theory proposes a mechanism by which the costs of underestimating a threat (e.g., injury or death) are deemed higher than overestimating threat (e.g., energy spent inefficiently, Haselton and Buss, 2000). Thus, this bias in costs of threat estimation supports a tendency to react (unnecessarily) fearful to potential threat and especially evolutionary relevant stimuli (Nesse, 2019). Considering that women used to be responsible for childcare and gathering food, they were possibly exposed to higher costs of underestimating threat for themselves and their offspring. Therefore, women may be more sensitive to fear of snakes, as a tendency to identity snakes as potential threat and reacting accordingly might have been evolutionary beneficial (Rakison, 2009).

Support for the evolutionary perspective explaining the sex ratio in anxiety comes from biological evidence for (neuro-)physiological differences in anxiety between women and men. Biological vulnerability factors enhancing anxiety in women have been reported, such as a higher physiological reactivity of the autonomic nervous system and of the hypothalamic–pituitary–adrenocortical (HPA) axis (Kelly et al., 2008; McLean and Anderson, 2009; Bangasser et al., 2010). Furthermore, structural and functional differences have been documented between men and women in regions relevant for the processing of fear and anxiety, such as the prefrontal cortex, the hippocampus, and the amygdala (Marques et al., 2016).

Considering the strong support for evolutionary and biological causes of sex differences in specific phobia, one might assume that fear of snakes is evolutionary hardwired (especially in women), implying that social influences do not play a significant role. However, it has been discussed that evolutionary and biological theories, e.g., differences in preparedness, only explain the ease and quantity of associative fear learning, but not whether associative fear learning takes place at all (Kawai, 2019). Thus, it cannot explain why some, but not all women, acquire fear of snakes (Frederikson et al., 1997), indicating that also other factors, such as socialization, may play a role. Multiple studies propose that genetic and environmental factors interact in the genesis of specific phobias (e.g., Ollendick et al., 2002; Loken et al., 2014; Sawyers et al., 2019). Thus, our finding that maternal (implicit) gender stereotypes influence girls’ fear of snakes and do not necessarily contradict evolutionary theories on sex differences in specific phobia. Possibly, the impact of parental gender stereotypes may be enhanced given a genetic (female) predisposition to fear responses. Vice versa the absence of parental gender stereotypes and encouragement of approach behavior in girls might alleviate biological influences on fear. However, determining the relative impact and interaction of genetic and social influences on sex differences in phobias, and snake phobia, specifically, goes beyond the scope of this study and has to be investigated in future research.

Beyond biological theories of fear, psychological models postulate a crucial role of associative learning for the acquisition of specific phobias. In general, three pathways for fear acquisition are assumed: classical conditioning, vicarious learning, and verbal threat information (Rachman, 1977). For influences of socialization on fear acquisition with special regard to gender differences, vicarious learning and verbal threat information, as well as reinforcement of specific behaviors, may be relevant (Ollendick and King, 1991; Möller et al., 2015). For these paths, at least up to school age, parents most likely play a prominent role in conveying verbal threat information, in terms of role modeling and also by reinforcing children’s anxious behavior.

Several studies including children of different ages suggest that both (fear-relevant) modeling behavior (Askew and Field, 2007) and verbal threat information (Muris and Field, 2010) can (differentially) affect anxiety levels in children (e.g., Remmerswaal et al., 2013). For example, a study with child–mother dyads was conducted where children observed their mothers’ positive or negative vocal and facial expressions in response to a toy snake or spider. When confronted with the toys, children‘s fear and avoidance responses were significantly enhanced after a negative response from the mother, with the effect being greater for girls than boys (Gerull and Rapee, 2002). To investigate effects of threat information on childhood fears, pictures of unfamiliar animals were presented to a children sample. Each picture was accompanied by positive, negative, or neutral information about the unknown animal. Implicit and self-reported fear as well as avoidance behavior increased when children were provided with negative information and decreased with positive information about the animals (Field and Lawson, 2003). With respect to gender differences, girls tend to report more incidences of informational learning as source of their fears than boys (Ollendick and King, 1991). These differences may reflect extant socialization practices and/or real differences in fear acquisition. Although girls do not seem to be more sensitive to informational threat learning in general, they were found to be especially susceptible to ambiguous threat information (Muris and Field, 2010). In sum, these differences in social fear learning between girls and boys may reflect biological (shown in animal research) or acquired differences in the impact of social information or in fear acquisition (as shown for fear conditioning paradigms, see Day and Stevenson, 2020).

Again, our results do not contradict previous evidence on the impact of associative learning on sex differences in specific phobia. Rather, especially vicarious and informational learning provides channels for communicating (implicit) gender stereotypes. Also, the fact that girls are only more susceptible to (ambiguous) information learning is well in line with our finding that implicit, but not explicit maternal gender stereotypes influence girls’ fear of snakes. Possibly, implicit gender stereotypes about girls’ expected fear reaction may mainly provide, of nature, indirect or ambiguous information. However, our results do not provide causal insights into these processes. Thus, it would be interesting to investigate whether girls are more susceptible to vicarious and/or informational threat learning *per se*, or whether parental gender stereotypes mediate these effects.

## Further Strengths and Limitations

In addition to the main findings, our correlational results indicate initially that mothers’ fear of snakes is significantly associated with their daughters’ fear of snakes. This stands in line with findings that enhanced anxiety of parents can be related to phobic and anxiety disorders in children (Muris et al., 1996; Ollendick and Horsch, 2007). However, opposite to our expectations, maternal fears did not explain additional variance of daughters’ fears when implicit stereotypes were considered in the regression model. This is surprising considering consistent evidence on the relationship between parental anxiety and children’s anxiety. For example, this relationship was shown between children and *both* parents, whereas other studies showed that fearfulness of the children was specifically related to their mothers’ fearfulness (Muris et al., 1996; Murray et al., 2009). Furthermore, this relationship was modulated by model learning (Muris et al., 1996). However, some studies show no association between children‘s performance on a behavioral approach test and parental phobic anxiety (Ollendick et al., 2012; van der Bruggen and Bögels, 2012). So far, no study has taken gender role stereotypes into account while investigating the relation between parental and children‘s anxiety – therefore, it is crucial to further elucidate this relationship with additional consideration of factors as, for example, gender roles and stereotypes.

Also, in line with the literature, we found a significant negative association between masculinity and trait anxiety for the mothers. That masculine traits have a diminishing effect on anxiety has been consistently reported in the literature (Arrindell, 2000; Muris et al., 2005; Stoyanova and Hope, 2012). Although there was no association between femininity and fear in our adult sample, this is also in line with a large part of the literature, in which associations between fear and masculinity are reported more frequently (Arrindell, 2000; Moscovitch et al., 2005) than between femininity and fear or both (Tucker and Bond, 1997).

Also for children, the literature consistently reports positive associations between fear and femininity and negative associations between fear and masculinity for non-clinical samples (Muris and Field, 2010) and children with anxiety (Ginsburg and Silverman, 2000; Muris et al., 2005). Unexpectedly, we could not find any support for this correlation in our study. A possible explanation could be that we only studied girls, whereas other studies considered mixed-gender samples, and thus, we might have limited variance in those constructs. In addition, due to our sample size, the statistical power to detect small-to-moderate effects may not be sufficient. Also, the reported associations appeared in much larger samples (see Ginsburg and Silverman, 2000; Muris et al., 2005). Based on power analyses of the given effect sizes in our studies, we recommend a sample size of at least *n* = 60 for future studies investigating associations between fear and gender variables. Furthermore, it seems important to investigate these relationships in more heterogeneous samples.

In contrast to the literature, we found no significant association between gender roles and stereotypes of mothers and their daughters neither for implicit nor for explicit measures. A possible explanation could be the relatively young age of our child sample because existing associations were shown mainly between parents and older children or adolescents (Ex and Janssens, 1998; Tenenbaum and Leaper, 2002). Furthermore, similarities are maybe not as strong as assumed because children are also exposed to other than parental influences that shape their views and attitudes (Martin et al., 2002). Thus, it is plausible that several studies, including our study, find no or only moderate correlations (Tenenbaum and Leaper, 2002).

Regarding our methodical approach, the association between children’s snake fear and their fear ratings with their approach behavior suggests that the BAT is suitable to measure fear on a behavioral level even in younger children (see also Klein et al., 2011). The importance of the multidimensional assessment of anxiety is supported by findings that self-reported anxiety does not always correspond to actual behavior in fearful situations (Cartwright-Hatton et al., 2003; Alpers and Sell, 2008). Moreover, self-reports reflect more controlled processes, whereas behavioral fear responses are more automatic – especially in children (Bijttebier et al., 2003; Strack and Deutsch, 2004). Thus, the assessment of behavioral components in addition to self-report can more adequately address the different components of fear (Ollendick et al., 2011) and may help to reduce the influence of response tendencies. This might be of specific importance in the research field of gender roles and sex differences because there is evidence that the fear ratings of men can be affected by conformation to the traditional male gender role (Pierce and Kirkpatrick, 1992). However, a ceiling effect occurred in the BAT. Of 38 girls, six did not approach the snake to the last step, raising the question whether the test did not provoke sufficient anxiety or whether the sample consists of low (snake) fearful girls. The latter assumption is supported by low-to-medium fear reports in the questionnaires. Furthermore, the courageous approach behavior of girls could also be explained by the presence of a female experimenter. Thus, the girls were not alone during the task and possibly felt encouraged by the (female) experimenter, who might have acted as a role model. This consideration is supported by the finding that positive modeling in a new situation can prevent the acquisition of fear (Egliston and Rapee, 2007).

Another important limitation of our study is that – due to its explorative character – we did not correct for multiple testing (see Streiner and Norman, 2011). Thus, we can only provide first evidence of a plausible relationship between maternal stereotypes and children’s fear. Of course, this needs to be replicated in further studies.

For practical reasons, we focused on mothers and their daughters, but we are aware that the father’s influence certainly plays a role as well. While mothers on average spend most time with their children (Lamb, 2000) and are thought to be the primary mediator of gender role attitudes for their offspring, it was also found that more masculine fathers have children with less feminine traits (Ex and Janssens, 1998). Similarly, children have less stereotypical attitudes toward their own gender when their fathers take on more household tasks (Turner and Gervai, 1995). Fathers also strongly influence children’s gender stereotypes of academic performance (Tomasetto et al., 2015). There is further evidence from a meta-analysis that fathers differentiate between sons and daughters more strongly than mothers (Lytton and Romney, 1991). In addition, fathers are more likely to reinforce exploratory and physical play in boys than in girls and expect more discipline from sons. These findings suggest that fathers also have an important influence on their children’s development that should not be neglected. However, the influence on children’s gender role orientation and gender stereotypes (Tenenbaum and Leaper, 2002) as well as on fear and anxiety (Möller et al., 2015) is likely to be an interaction of both parents. Therefore, it would be relevant for future studies to investigate the influence of maternal and paternal characteristics on childhood anxiety of girls, but also of boys (Salcuni et al., 2015).

Finally, it is very important to note that the study of maternal or generally parental influences on children’s behavior is not about assigning blame but should help to identify relevant risk as well as protective factors. It can be assumed that parents (and other primary caregivers) do not want to influence their children willingly in an unfavorable way, but rather want to do the best for their children. In addition, there is evidence that gender roles and possibly gender role stereotypes also have genetic/biological components and thus cannot be understood exclusively as a result of socialization (e.g., Polderman et al., 2018). Nonetheless, the identification of potential risk factors offers the opportunity for parents to reflect on their behavior and to expand their scope of action. Furthermore, it can be useful to consider these aspects in prevention and treatment programs that take parents into account (Wei and Kendall, 2014).

## Summary and Future Direction

In sum, the present study replicated and extended links between stereotypes and fear. For one, masculinity and anxiety are negatively correlated in mothers. Also, we found mothers’ and their daughters’ specific fears to be associated. Most importantly, for the first time, we showed that implicit gender stereotypes of mothers are associated with daughters’ specific fear and their fear in presence of a fear-relevant animal. Interestingly, maternal fears did not predict daughters’ fears beyond implicit gender stereotypes. As this is one of the few experimental studies examining the relationship between parental gender stereotypes and children’s fear, it may motivate replications with larger and more heterogeneous samples including fathers and sons and a wider range of possible fear domains. Increasing awareness of gender stereotypes may be a promising approach to prevent fears and phobias in girls and to establish targeted treatment modalities for women.

## Data Availability Statement

The data supporting the conclusions of this article are available on request in the MADATA - Research Data Repository of the University of Mannheim (doi: 10.7801/388), available at: https://madata.bib.uni-mannheim.de/.

## Ethics Statement

The studies involving human participants were reviewed and approved by Ethics Committee at the University of Mannheim (EK Mannheim 08/2018). Written informed consent to participate in this study was provided by the participants’ legal guardian/next of kin.

## Author Contributions

AG and GA contributed to research idea, design, and methodology. AG, L-AF, and MB performed data preparation and data analysis and were involved in manuscript writing and preparation. MB collected the data. All authors contributed to the article and approved the submitted version.

## Funding

The publication of this article was funded by the Ministry of Science, Research and the Arts Baden-Württemberg and the University of Mannheim.

## Conflict of Interest

The authors declare that the research was conducted in the absence of any commercial or financial relationships that could be construed as a potential conflict of interest.

## Publisher’s Note

All claims expressed in this article are solely those of the authors and do not necessarily represent those of their affiliated organizations, or those of the publisher, the editors and the reviewers. Any product that may be evaluated in this article, or claim that may be made by its manufacturer, is not guaranteed or endorsed by the publisher.

## Footnotes

^1^AIP data for one child were excluded due to technical problems.
